# Sensing with (One or Many) Upconverting Nanoparticles

**DOI:** 10.1021/acs.accounts.5c00896

**Published:** 2026-04-30

**Authors:** Fernando E. Maturi, Erving Ximendes, Antonio Benayas, Dirk H. Ortgies, Emma Martín Rodríguez, Patricia Haro-González, Daniel Jaque

**Affiliations:** † Nanomaterials for Bioimaging Group, Departamento de Física de Materiales, Facultad de Ciencias, 16722 Universidad Autónoma de Madrid, 28049 Madrid, Spain; ‡ Nanomaterials for Bioimaging Group, Instituto Ramón y Cajal de Investigación Sanitaria, Hospital Ramón y Cajal, 28034 Madrid, Spain; § Instituto de Ciencia de Materiales Nicolás Cabrera, Universidad Autónoma de Madrid, 28049 Madrid, Spain; ∥ Institute for Advanced Research in Chemical Sciences, Universidad Autónoma de Madrid, 28049 Madrid, Spain; ⊥ Nanomaterials for Bioimaging Group, Departamento de Física Aplicada, Universidad Autónoma de Madrid, 28049 Madrid, Spain

## Abstract

Lanthanide-doped upconverting nanoparticles (UCNPs) convert near-infrared (NIR) light into visible emission through multiphoton absorption processes. This property has made them useful tools for sensing and single-particle studies in biological environments. Their characteristic narrow emission lines, resistance to photobleaching, and the thermal coupling between the excited states of trivalent erbium (Er^3+^) have positioned UCNPs as a practical platform for intracellular thermometry despite their moderate sensitivity. Their response can be measured with standard microscopes using inexpensive NIR excitation sources, enabling experiments that are not easily achieved with other types of nanoscale thermometers. However, using UCNPs inside living cells also reveals important limitations because the intracellular medium is chemically complex and may alter the emission properties that form the basis of the thermal readout. Variations in local pH, ion concentration, viscosity, or molecular crowding can shift energy levels or modify nonradiative relaxation pathways. As a result, establishing the reliability of UCNP thermometry inside cells requires dedicated studies on particle stability, surface chemistry, and the influence of the cytoplasmic environment. Understanding these effects is essential for determining whether ratiometric UCNP thermometry can serve as a quantitative intracellular tool rather than only a qualitative indicator of heating. UCNPs have also enabled new single-particle experiments through optical trapping. A tightly focused NIR beam can immobilize an individual nanoparticle while simultaneously exciting its upconversion emission, allowing continuous monitoring of spectral changes, rotational dynamics, and local mechanical properties. These studies provide access to information that is hidden in ensemble measurements, such as particle–particle radiative interactions or the coupling between particle rotation/movement and local viscosity. Yet optical trapping also brings its own challenges: the forces acting on sub-100 nm UCNPs are modest, and trapping stability is strongly affected by laser-induced heating. Increasing dopant concentration improves confinement but raises thermal load, leading to a trade-off that must be carefully balanced. Approaches based on plasmonic enhancement are not suitable due to their additional heating, motivating interest in alternative photonic structures such as dielectric metasurfaces that could strengthen confinement while keeping temperatures low. Together, intracellular thermometry and single-particle trapping illustrate both the strengths and the current limitations of UCNPs. Their optical properties make them accessible probes for measuring local temperature and mechanical responses, but their performance heavily depends on the surrounding environment. In this Account, we summarize our efforts to understand UCNP behavior under realistic biological and trapping conditions. We discuss the stability of the thermometric response inside cells, the confinement–heating balance in optical traps, and the opportunities offered by single-particle measurements for studying light–matter interactions at the nanoscale. These considerations outline a path toward more reliable sensing and manipulation strategies based on UCNPs.

## Graphical abstract

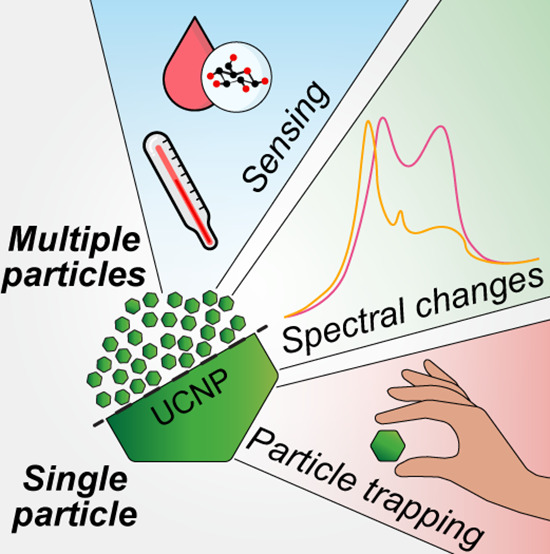

## Key References

-

Vetrone, F. ; Naccache, R. ; Zamarron, A. ; Juarranz de la Fuente, A. ; Sanz-Rodriguez, F. ; Martinez Maestro, L. ; Martin Rodriguez, E. ; Jaque, D. ; Garcia Sole, J. ; Capobianco, J. A. Temperature sensing using fluorescent nanothermometers. ACS Nano 2010, 4, 3254–3258 20441184 10.1021/nn100244a.^1^ Upconverting nanoparticles (UCNPs) enable temperature measurement within a living cell from 25 °C to the thermally induced death temperature of 45 °C, demonstrating the versatility of upconversion for thermal sensing and imaging.
-

Labrador-Páez, L. ; Pedroni, M. ; Speghini, A. ; Garcia-Sole, J. ; Haro-Gonzalez, P. ; Jaque, D. Reliability of rare-earth-doped infrared luminescent nanothermometers. Nanoscale 2018, 10, 22319–22328 30468230 10.1039/c8nr07566b.^2^ Lanthanide-based nanoparticles experience spectral distortions caused by environmental and experimental factors, such as absorption by the surrounding media and self-absorption of the nanoparticle, requiring careful attention to ensure accurate thermal sensing using UCNPs.
-

Rodríguez-Sevilla, P. ; Rodríguez-Rodríguez, H. ; Pedroni, M. ; Speghini, A. ; Bettinelli, M. ; Solé, J. G. ; Jaque, D. ; Haro-González, P. Assessing Single Upconverting Nanoparticle Luminescence by Optical Tweezers. Nano Lett. 2015, 15, 5068–5074 26120948 10.1021/acs.nanolett.5b01184.^3^ The optical trapping of a single trapped UCNP reveals a change in the spectral emission profile in comparison to an ensemble of trapped UCNPs due to self-absorption coming from particle–particle interactions, which should be considered for sensing applications.
-

Rodríguez-Sevilla, P. ; Zhang, Y. ; de Sousa, N. ; Marques, M. I. ; Sanz-Rodriguez, F. ; Jaque, D. ; Liu, X. ; Haro-González, P. Optical Torques on Upconverting Particles for Intracellular Microrheometry. Nano Lett. 2016, 16, 8005–8014 27960460 10.1021/acs.nanolett.6b04583.^4^ Adequate interpretation of the polarized emission generated by an optically trapped UCNP within a live cell makes it possible to access its rotation dynamics and the intracellular viscosity.

## 1 Introduction

Since Auzel’s seminal description of the upconversion process in 1966,^5,6^ upconverting nanoparticles (UCNPs) have become an attractive material for sensing and imaging due to their unique ability to convert multiple lower-energy near-infrared (NIR) photons into high-energy ultraviolet (UV) or visible (Vis) emissions. While the field originally focused on incorporating lanthanide ions, such as Yb^3+^ and Er^3+^, into glass^7–11^ and fiber-based systems,^12,13^ its expansion was catalyzed by exploring the intrinsic temperature-sensing capability of Er^3+^,^7,8^ and advancing nanomaterial synthesis strategies, which enabled better control over particle size, composition, and optical performance.^14–17^ Beyond their early adoption in photodynamic therapy (PDT),^18,19^ these advances expanded the portfolio of UCNPs, which have since found applications in optoelectronic devices,^20^ optogenetics,^21^ super-resolution,^22,23^ photothermal therapy (PTT),^24^ drug delivery,^25^ biomolecule detection,^26^ live-cell imaging,^27^ single-particle tracking,^28^ and solar cells,^29^ to mention a few.

UCNPs have demonstrated excellent performance in the realm of (bio)­sensing due to the simplicity of their operating principles, resulting in an optical response that is sensitive to changes in the local environment, with a significant impact on both fundamental research and practical applications. However, to truly understand UCNPs’ potential, we must distinguish between two distinct ways of using them: the ensemble range, with thousands of nanoparticles emitting light simultaneously, and the single-particle regime, which is a challenging task due to the faint signal coming from a single nanoparticle. Therefore, the main milestones of the field come from achievements bridging these two regimes:

- Temperature sensing inside cells: UCNPs have enabled precise luminescence thermometry within the physiological range, allowing researchers to monitor temperature changes that are critical for distinguishing between cellular life and death.
- Environmental and experimental perturbations in luminescence nanothermometry: UCNPs have made it possible to perform a detailed analysis on how environmental and experimental factors influence the reliability of temperature readings. Perturbations arise both from optical absorption in the surrounding medium and from self-absorption by the UCNPs themselves, necessitating careful consideration for accurate thermal sensing.
- Spectral differences between single and ensemble UCNPs: Comparing the spectral fingerprints of ensembles versus single UCNPs reveals distinct differences due to interparticle interactions and self-absorption. Advances in optical trapping have been instrumental in enabling the measurement and quantification of these differences, as researchers can now observe luminescence transitions by incrementally increasing the number of working UCNPs from one to many.
- Intracellular sensing with an optically trapped UCNP: A combination of optical trapping and single particle spectroscopy enables real-time monitoring of single-particle rotation in live cells through polarized emission, allowing remote measurement of parameters like viscosity.

These four achievements provided key insights into how luminescence experiments based on UCNP signals should be performed and interpreted, playing an important role in advancing the field. More importantly, the knowledge gained from these studies has become standard practice for designing and deploying UCNPs in various sensing applications, extending beyond thermal sensing to other types of luminescence-based measurements. By revisiting these developments, this Account traces the evolution of the field and highlights contributions from our group that continue to guide ongoing advances in luminescence sensing using both ensembles and single UCNPs.

## 2 Sensing with Upconverting Nanoparticles

The success of UCNPs in sensing comes from their ability to convert NIR excitation into high-energy UV/Vis emissions through multiphoton absorption. This mechanism is particularly advantageous for biological applications, as NIR light is weakly absorbed by biological media, ensuring deep tissue penetration with minimal background interference.^30^ By using lanthanide ions to fabricate UCNPs, the resulting narrow and long-lived emission bands are easily distinguished from background signals and respond sensitively to changes in pH, temperature, and medium composition, providing valuable chemical and physical insights into their surroundings.^31^

Sodium yttrium fluoride codoped with Yb^3+^/Er^3+^ (NaYF_4_:Yb^3+^/Er^3+^) is the most employed material in the synthesis of UCNPs because the NaYF_4_ host matrix is chemically stable, transparent to excitation and emission wavelengths, and exhibits low phonon energies (ca. 360 cm^–1^), minimizing nonradiative losses and maximizing luminescence efficiency.^32,33^ Its thermometric mechanism can be triggered upon NIR excitation via Yb^3+^ using low-power and inexpensive 980 nm laser diodes, achieving green upconversion emission from the thermally coupled levels of Er^3+^, ^2^H_11/2_, and ^4^S_3/2_ (Figure 1a).^34^ Because the energy gap (Δ*E*) between these levels is small, their populations follow Boltzmann statistics:^35^

1 $$LIR=\frac{I_{525}}{I_{545}}=C\;\exp\left(\frac{-\Delta E}{k_{B}T}\right)$$

where *LIR* is the luminescence intensity ratio between the integrated intensities of the ^2^H_11/2_ → ^4^I_15/2_ (*I*_525_) and ^4^S_3/2_ → ^4^I_15/2_ (*I*_545_) transitions of Er^3+^, *C* accounts for the degeneracy and spontaneous emission rates, *k*_*B*_ is the Boltzmann constant, *T* is the absolute temperature, and Δ*E* typically lies in the 650–750 cm^–1^ range.^36^ This self-referenced thermometric approach has become widespread due to its robustness and simple calibration, making NaYF_4_:Yb^3+^/Er^3+^ and similar upconverting materials attractive for luminescence-based thermal sensing.

**Figure 1:**
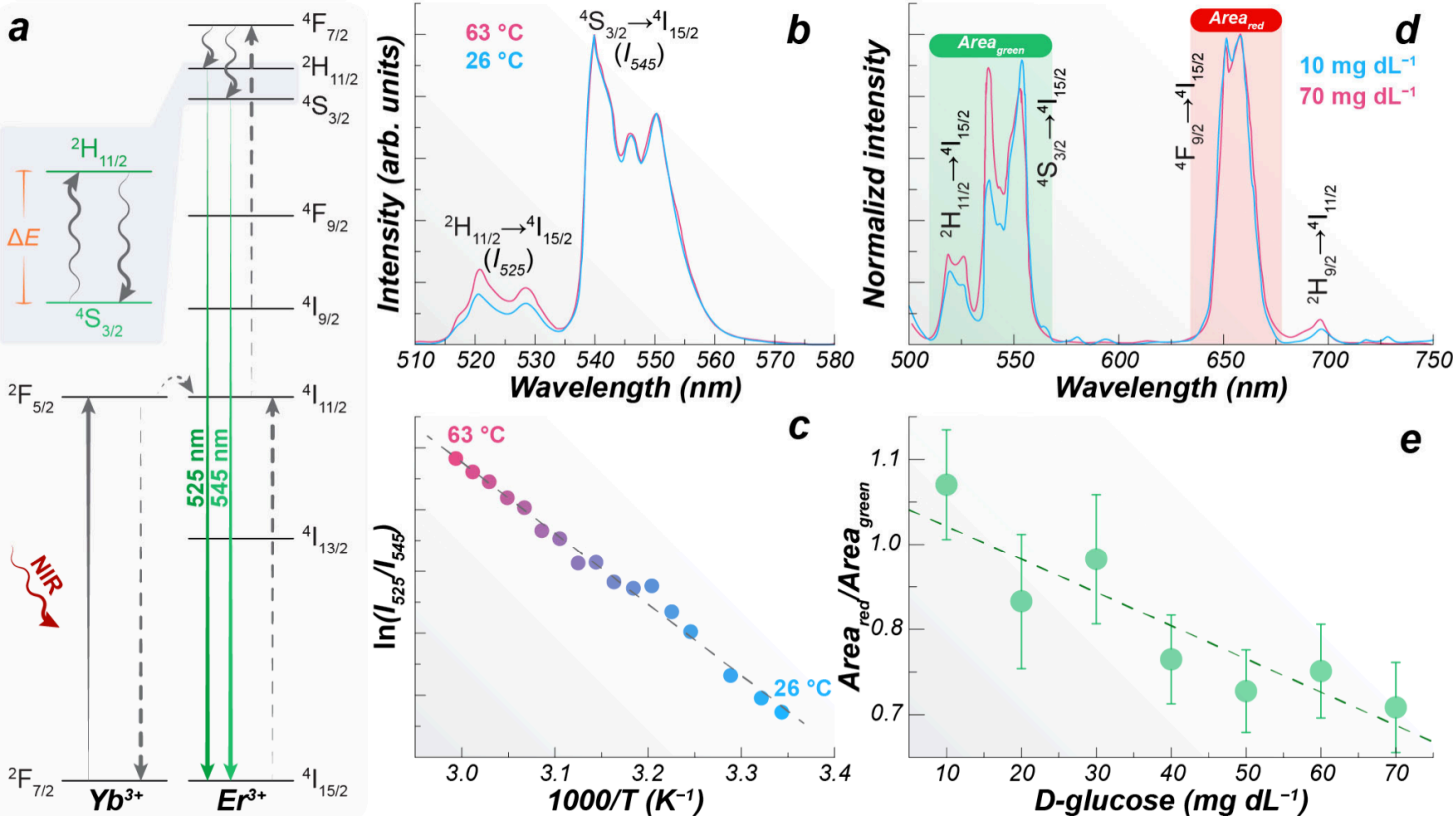
(a) Partial energy level diagram of Yb^3+^/Er^3+^, (b) upconversion emission spectra (920 nm excitation), and (c) thermometric calibration of NaYF_4_:Yb^3+^/Er^3+^ UCNPs. (d) UCNP emission in the presence of d-glucose. (e) Red-to-green ratio calibration as a function of d-glucose. (b, c) Adapted from ref 1, with permission from the American Chemical Society, Copyright 2010. (d, e) Adapted from ref 48, with permission from Elsevier, Copyright 2025.

### 2.1 Thermometry Inside Cells

Temperature regulates fundamental cellular activities, providing a direct pathway to distinguish between healthy and diseased (e.g., cancer) cells based on subtle thermal variations. While conventional thermometers lack the required spatial resolution to operate within the complex cellular environment, nanotechnology has provided a powerful solution through luminescence nanothermometry.^37^ By harnessing the temperature-dependent light emission of UCNPs, it is possible to perform remote temperature measurements within living cells. This idea was first elegantly demonstrated in our group by Vetrone et al., employing polyethylenimine-capped NaYF_4_:Yb^3+^/Er^3+^ UCNPs as thermal nanoprobes in both liquids and HeLa cervical cancer cells.^1^ The resulting UCNPs (mean diameter of 18 nm) displayed upconversion emission following the Boltzmann law (eq 1) across the 26–63 °C temperature range (Figure 1b), also enabling easy internalization into immortalized human carcinoma cells after 1.5 h incubation via endocytosis.^38^ Using a confocal microscope and a metallic platform equipped with a resistance heater, optical and fluorescence images of the cells were acquired at varying temperatures. By analyzing the *LIR* from the fluorescence images (Figure 1c), the heater voltage was converted into a precise measurement of the cellular media temperature, observing cellular death at 45 °C, which was corroborated by the visual presence of a small membrane fragment in the corresponding transmission optical image.

By taking advantage of their physicochemical stability and resistance to photobleaching upon prolonged light exposure,^39^ UCNPs were then established as an important tool for intracellular thermal sensing. While initial progress was hindered by concerns regarding cytotoxicity and specific cellular delivery, subsequent research demonstrated that proper surface functionalization is an effective strategy to enhance biocompatibility and facilitate *in situ* organelle targeting.^40,41^ The lessons learnt from this pioneering study served as stepping stones to build the foundational knowledge for modern thermal mapping,^1^ which is currently used for selective labeling of cell membranes and the precise targeting of lysosomes and mitochondria,^42,43^ reinforcing the suitability of Yb^3+^/Er^3+^-based UCNPs as thermal probes within the complex environment of the living cell.

### 2.2 Biosensing

Although most studies employing UCNPs for sensing are dedicated to measuring temperature, there are also applications as a biosensor for pH or various analytes that interact with their upconversion luminescence.^44,45^ A recent example from our group employs a quenching mechanism based on the inner filter effect of the upconversion emissions of the Yb^3+^/Ho^3+^ pair to study Cu^2+^ concentrations in aqueous solutions.^46^ This field is ever-expanding in parallel with the growing understanding of the underlying principles in UCNPs as introduced up to now. One important example with biological implications is the detection of glucose levels in bioliquids due to the increasing prevalence of diabetes. The vibrations of hydroxyl groups are known quenchers of the Er^3+^ ion, and sugars present several of them. This affects the population of the excited states of Er^3+^ and, consequently, their upconversion emission, enabling UCNPs to be used as a glucose sensor.^47^ Recently, López-Peña et al. showed that the upconversion emission of the Yb^3+^/Er^3+^ pair can also be used to detect glucose in solution in tear fluids, using a phenylboronic acid-modified porous silicon substrate and paper substrates (Figure 1d,e).^48^

A different type of quenching mechanism that exploits the principles of Förster resonance energy transfer was explored by Yao et al. in our group for hypoxia sensing.^49^ A nonfluorescent azo dye was bound to the UCNP’s surface and enabled quenching of the upconversion emission. In a biological hypoxia/anoxia environment, enzymes are upregulated, and the azo-bond is reductively cleaved, stopping the quenching of the upconversion and enabling the recovery of the UCNP’s emissions, which thereby act as the sensor of hypoxia (Figure 2a,b). This example demonstrates another advantage of UCNPs: depending on their dopant combination, such as NaGdF_4_:Nd^3+^/Yb^3+^/Tm^3+^, these nanoparticles can produce strong and long-lived infrared emissions in addition to their upconversion luminescence.^50^ Together with a shift in excitation wavelengths, this paves the way for improved biomedical applications.^51–53^

**Figure 2:**
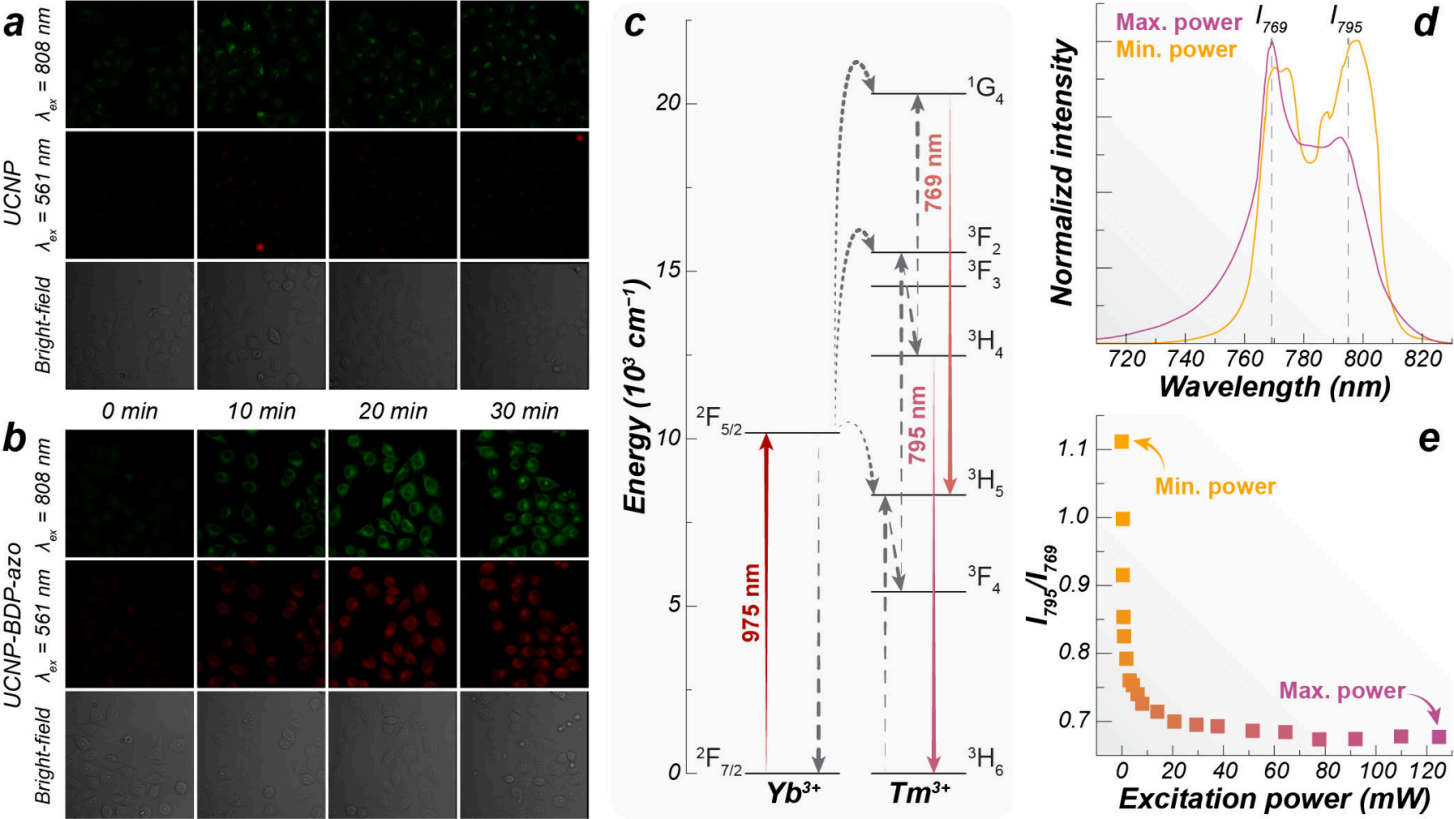
Confocal images displaying the hypoxic response in sealed HeLa cell cultures of (a) NaGdF_4_:Nd^3+^/Yb^3+^/Tm^3+^ UCNPs and (b) UCNP-BDP-azo. (c) Partial energy level diagram of Yb^3+^/Tm^3+^, (d) upconversion emission spectra of SrF_2_:Yb^3+^/Tm^3+^ luminescent thermometers (975 nm excitation), and (e) power-dependent intensity ratio changes. (a, b) Reproduced from ref 49, with permission from the Royal Society of Chemistry, Copyright 2025. (d, e) Adapted from ref 2, with permission from the Royal Society of Chemistry, Copyright 2018.

### 2.3 Artifacts

The ability to perform remote temperature sensing, together with the flexibility of these materials for chemical detection, motivated the adoption of UCNPs for luminescent thermometry^54^ and point-of-care devices.^55^ Given their promise for applications in nanomedicine and microelectronics, UCNP-based thermometers were then rapidly adopted by the community, often without sufficient critical validation. The limitations of this approach soon became evident, as temperature measurements with UCNPs proved less reliable than initially assumed.^56,57^ In many cases, spectral distortions induced by variations in excitation power^58^ or by emission from energy levels other than the thermally coupled Er^3+^ states^59^ led to misleading temperature readouts.

These issues were not exclusive to UCNPs but reflected a broader problem in luminescence thermometry. This realization motivated our group to conduct the first systematic investigation into how experimental and environmental factors can distort emission spectra used for temperature determination. In the study by Labrador-Páez et al.,^2^ several key sources of error were identified, including changes in emission band shape with excitation power density, self-absorption of emitted light by the nanothermometers themselves, and reabsorption of luminescence by the surrounding medium, such as solvents or biological tissue. Rather than simply reducing signal intensity, these effects modify the spectral profile, biasing ratiometric approaches that assume stable intensity ratios. As a consequence, apparent temperature variations can be observed even when the actual thermal conditions remain unchanged, highlighting the need for careful experimental design and validation in luminescence thermometry.

Excitation power artifacts arise because different emitting levels may follow distinct power-dependent dynamics, such as those coming from ^3^H_4_ → ^3^H_6_ (795 nm, two photon excitation) and ^1^G_4_ → ^3^H_5_ (769 nm, three photon excitation) in Tm^3+^ (Figure 2c). Therefore, using an intensity ratio combining these two emission bands can result in deviations up to 49% mW^–1^ (Figure 2d,e). Experiments with SrF_2_:Yb^3+^/Tm^3+^ UCNPs showed that, even at constant laser power, depth-dependent changes in excitation density systematically modify emission intensity ratios. This led to false temperature variations, with apparent thermal equivalent noise (TEN) reaching 28.7 °C mm^–1^ for the analyzed Tm^3+^ band, and up to 46.7 °C mW^–1^ for other systems. These artifacts are amplified when using high-numerical-aperture (NA) objectives, making them particularly relevant for intracellular thermometry.

Self-absorption artifacts originate from the reabsorption of emitted photons by neighboring nanoparticles due to spectral overlap between absorption and emission bands. Nd^3+^-based thermometers, widely used for their strong NIR emission, are especially vulnerable. Increasing the optical path length within a colloid distorts the spectral profile, producing depth-dependent changes unrelated to temperature and errors up to 26 °C mm^–1^. This effect becomes more significant at high nanoparticle concentrations or long optical paths, conditions commonly found in tissues, microfluidic devices, and aggregated intracellular environments. Environmental absorption poses the most severe challenge for biological applications. Water strongly absorbs near 1300 nm, overlapping the Nd^3+^ emission at 1320 nm. As luminescence propagates through aqueous media, selective absorption reshapes the spectrum, yielding TEN values up to 92 °C mm^–1^. Under these conditions, depth variations of only 0.2 mm in vitro can cause temperature errors approaching 15 °C, while in vivo measurements can deviate by tens of degrees.^2^

These effects proved to be only the tip of the iceberg. For example, the presence of ions such as Fe^2+/3+^ in aqueous media can induce temperature deviations greater than 10 °C when silver sulfide nanoparticles are used as thermal probes.^60^ In biological media, the situation becomes even more complex because cellular activity can alter the local environment of a luminescent thermometer, introducing variability in its optical response. Such cell-induced changes have been clearly observed in studies with green fluorescent protein, one of the most widely used luminescent materials for bioimaging and sensing.^61^ Taken together, these effects demonstrate that there is still a long way to go before luminescent thermometers can be used for reliable in vivo measurements, mainly because light traveling through tissues encounters several obstacles, compromising spectral shape and, consequently, spectral temperature readouts.^62^

These photo- and chemically induced sources of bias have encouraged the community to explore alternative strategies for luminescence thermometry. In particular, approaches that do not rely exclusively on Er^3+^ emission or on ratiometric intensity measurements have gained attention. Lifetime-based methods, for instance, are generally less sensitive to intensity distortions and environmental absorption effects, offering a more robust route for temperature determination under complex experimental conditions.^63–66^ In this sense, lifetime thermometry has proven to be a good strategy to circumvent tissue-induced spectral distortions, even allowing for probing microwave-induced brain heating using the lifetime of semiconductor nanoparticles.^67^ Relying on similar approaches is a crucial step to really achieve reliable temperature readouts because, by surveying the literature and mapping commonly used Ln^3+^, nearly all bands used for nanothermometry suffer from at least one major artifact, with only the Nd^3+^ emission near 1060 nm remaining unaffected.^2^

The main message here is that luminescence thermometry cannot be trusted without performing essential validation steps. To check for artifacts, those steps must include (i) spectral measurements at different excitation powers; (ii) depth-dependent assessments to evaluate self-absorption; and (iii) calibrations accounting for environmental absorption. Additional strategies include increasing thermal sensitivity to reduce TEN, developing more efficient emitters and detectors, and optimizing optical systems. This is fundamental for providing critical corrections for the field, demonstrating that luminescent nanothermometers are not intrinsically reliable, and that unaccounted artifacts can produce temperature errors far exceeding biologically relevant variations.

## 3 Optical Trapping

Optical Trapping (OT) is a remote, versatile, and noninvasive tool fundamental to modern nanoscience. This Nobel Prize-winning technique enables three-dimensional (3D) manipulation of colloidal objects, from micro- to the nanoscale, allowing the isolation, controlled movement, and monitoring of individual particles, such as luminescent nanothermometers.^68^ Trapping relies on tightly focusing a laser beam using a high-NA objective, as shown in Figure 3a. The core OT mechanism involves optical forces generated by the focused beam, arising from momentum exchange between the beam light and the trapped particle or from the interaction between particle polarization and electric field gradients. These optical forces break down into two main components: the scattering force (*F*_*scat*_), acting along the beam propagation direction and pushing the particle forward (proportional to light intensity), and the gradient force (*F*_*grad*_), which pulls the particle toward regions of higher intensity (i.e., the center of the optical trap). To achieve stable 3D trapping, the axial gradient force, which draws the particle into the focus, must be strong enough to overcome the scattering force that pushes the particle in the direction of the light (i.e., *F*_*grad*_ > *F*_*scat*_).^69^

**Figure 3:**
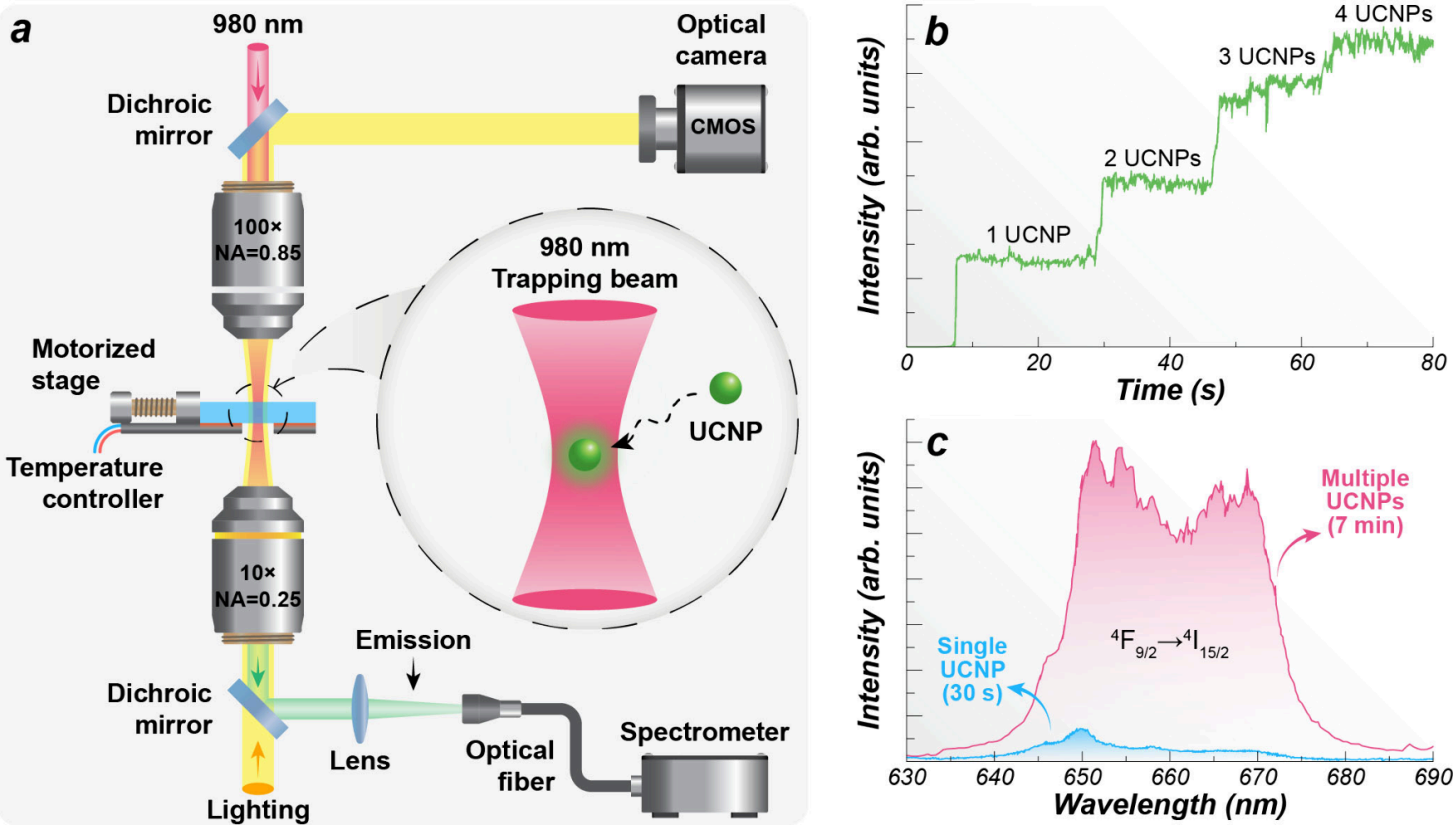
(a) Schematic of a typical optical trapping experimental setup used for UCNPs. (b) Increase in the emission intensity as more UCNPs get trapped under the excitation beam over time. (c) Emission spectra obtained from a single and multiple SrF_2_:Yb^3+^/Er^3+^ UCNPs after different irradiation times. (b, c) Adapted from ref 3, with permission from the American Chemical Society, Copyright 2015.

For nanoparticles whose size (*d*) is much smaller than the laser wavelength λ (*d* ≪ λ, in the Rayleigh regime), they behave as dipoles.^70^ In this regime, *F*_*grad*_ corresponds to the force a dipole experiences in a nonuniform electric field. At this scale, the optical force is mainly determined by the particle’s electronic polarizability, although it also strongly depends on electrostatic properties, such as the particle’s zeta potential, rather than just its volume. Trapping nanoparticles, especially for those where *d* < 100 nm, is challenging because the resulting optical forces are typically weak (usually below 0.01 pN for sub-100 nm UCNPs), and the trap potential is roughly equivalent to the system’s thermal energy.^71^ In addition, the nanoparticle’s Brownian motion, driven by thermal energy, significantly increases with temperature, making it easier for the particle to escape from the trap, where even a slight temperature rise can destabilize the particle confinement.^72^ Therefore, understanding the nanoparticle’s diffusion velocity, which strongly depends on temperature, is crucial for predicting its dynamics.

To overcome limitations in stability and force, researchers have developed two main strategies. The first approach relies on the surface modification, where, for instance, decorating UCNPs with thermoresponsive polymer coatings such as poly­(*N*-isopropylacrylamide) (PNIPAM) has achieved over 10-fold increase in optical force, enabling stable trapping up to 45 °C.^73^ The second key approach aims to reduce the trap volume. By using a photonic nanojet (PNJ) generated by an optically trapped dielectric microsphere to focus the laser beam beyond the diffraction limit, this process enhances the intensity gradient and, consequently, the trapping force (up to seven times the trap stiffness compared to conventional methods). Our group demonstrated how PNJs allow for stable trapping of UCNP nanoparticles as small as 8 nm across the entire liquid water temperature range (20–90 °C).^74^

### 3.1 Spectral Changes in Single-Trapped Nanoparticles

OT is an ideal platform for studying individual UCNPs due to the dual functionality of the tightly focused laser beam. In this scenario, a single NIR beam simultaneously generates the gradient forces required to trap and move the UCNPs and serves as the excitation source. This combination enables not only precise tracking of the UCNPs’ position but also provides the possibility of recording the luminescence signal generated by a controlled number of UCNPs (from single to many UCNPs).^75^ The sequential incorporation of UCNP into the optical trap allows the distinction between the signal generated by a single versus multiple UCNPs, evidencing the possible existence of particle–particle interactions.

A study carried out by Rodríguez-Sevilla et al. demonstrated this principle by introducing a method to stably immobilize and precisely localize strontium fluoride codoped with Yb^3+^/Er^3+^ (SrF_2_:Yb^3+^/Er^3+^) UCNPs dispersed in heavy water using 980 nm optical tweezers.^3^ The authors trapped a single UCNP (*d* = 8 nm), enabling the acquisition of a single-particle emission spectrum, a difficult task due to the Brownian motion taking place in colloidal suspensions. The isolated particle exhibited a dominant emission peak near 649 nm, corresponding to the Er^3+^ ^4^F_9/2_ → ^4^I_15/2_ transition. Under continuous irradiation, the emission intensity increased in a step-like way as more UCNPs were gradually trapped under the excitation beam (Figure 3b). With more UCNPs getting trapped, the emission spectra became broader with two comparable peaks at 650 and 665 nm (Figure 3c). These changes were attributed to radiative self-absorption (radiation trapping) occurring when photons emitted by one nanoparticle are reabsorbed by nearby UCNPs before detection, which is absent in the isolated particle but becomes relevant in ensembles confined within the trap. This work provided the first experimental evidence that interparticle radiative interactions can affect UCNP emission in colloidal suspensions, highlighting radiation trapping as an overlooked mechanism that must be considered when interpreting the optical response of UCNP ensembles.

Despite that, OT offers a major advantage for studying individual UCNPs because it reveals intrinsic photophysical properties that are often hidden in ensemble measurements. By isolating a single nanoparticle, researchers can examine effects such as excitation power dependence and anisotropy-related luminescence. This approach also enables detailed studies of photostability, emission dynamics, polarization effects, and interparticle energy transfer at the nanoscale.^76^ These capabilities expand sensing applications, including the detection of ions, biomolecules, and local environmental changes such as intracellular temperature and viscosity. Studying UCNPs at the single-particle level also provides deeper insight into energy transfer and upconversion mechanisms, ultimately improving the reliability and performance of luminescence-based sensing.^77^

### 3.2 Viscosity

Beyond single-particle spectroscopy, OT also provides direct access to the mechanical response of a nanoparticle interacting with its surrounding medium. A trapped UCNP behaves as a thermally driven harmonic oscillator, and its motion encodes information about local properties such as temperature, viscosity, and viscoelasticity. This principle underpins optical microrheology, which uses the motion of luminescent micro- or nanoparticles to extract local rheological parameters. Two operational modes exist: active and passive. Active approaches apply external forces, such as optical trapping or rotation, to drive particle motion, whereas passive methods rely on Brownian fluctuations to probe the mechanical response.^78^

Many biological materials are viscoelastic and can be described by the complex shear modulus *G*(ω) = *G*′(ω) + *iG*″(ω), where the real part reflects elastic energy storage and the imaginary part accounts for viscous dissipation. Optical microrheology provides different experimental routes to sample *G*′(ω) and *G*″(ω) over specific frequency ranges, set either by the imposed motion in active schemes or by the spectrum of thermal fluctuations in passive ones.^78^ UCNPs are well suited to both modes, as the same particle can be optically driven or allowed to fluctuate freely, with its luminescence providing a sensitive, background-free readout of position and orientation.

A representative example is the work of Rodríguez-Sevilla et al., who used the strong polarization dependence of the red Er^3+^ emission in hexagonal NaYF_4_:Yb^3+^/Er^3+^ UCNPs to turn a single optically trapped UCNP into a local viscosity probe.^4^ Polarized spectroscopy revealed how a nonspherical UCNP orients in a linearly polarized trap (Figure 4a–c). After proper calibration, the experimental determination of the emission intensity ratio *I*_656_/*I*_664_ allowed for the remote determination of the orientation of the UCNP within the optical trap. This, in turn, allowed authors to map the torque landscape and link the particle’s rotational dynamics to the surrounding viscosity. The transition time for a 90° rotation of the UCNP within the trap, extracted in real time from spectral changes, yielded access to the value of local viscosity. Inside HeLa cells (Figure 4d,e), both active torque-driven rotation and passive analysis of angular fluctuations revealed a mean cytoplasmic viscosity of ∼2.5 Pa s, decreasing with rotation frequency, consistent with a viscoelastic, liquid-like cytoplasm.

**Figure 4:**
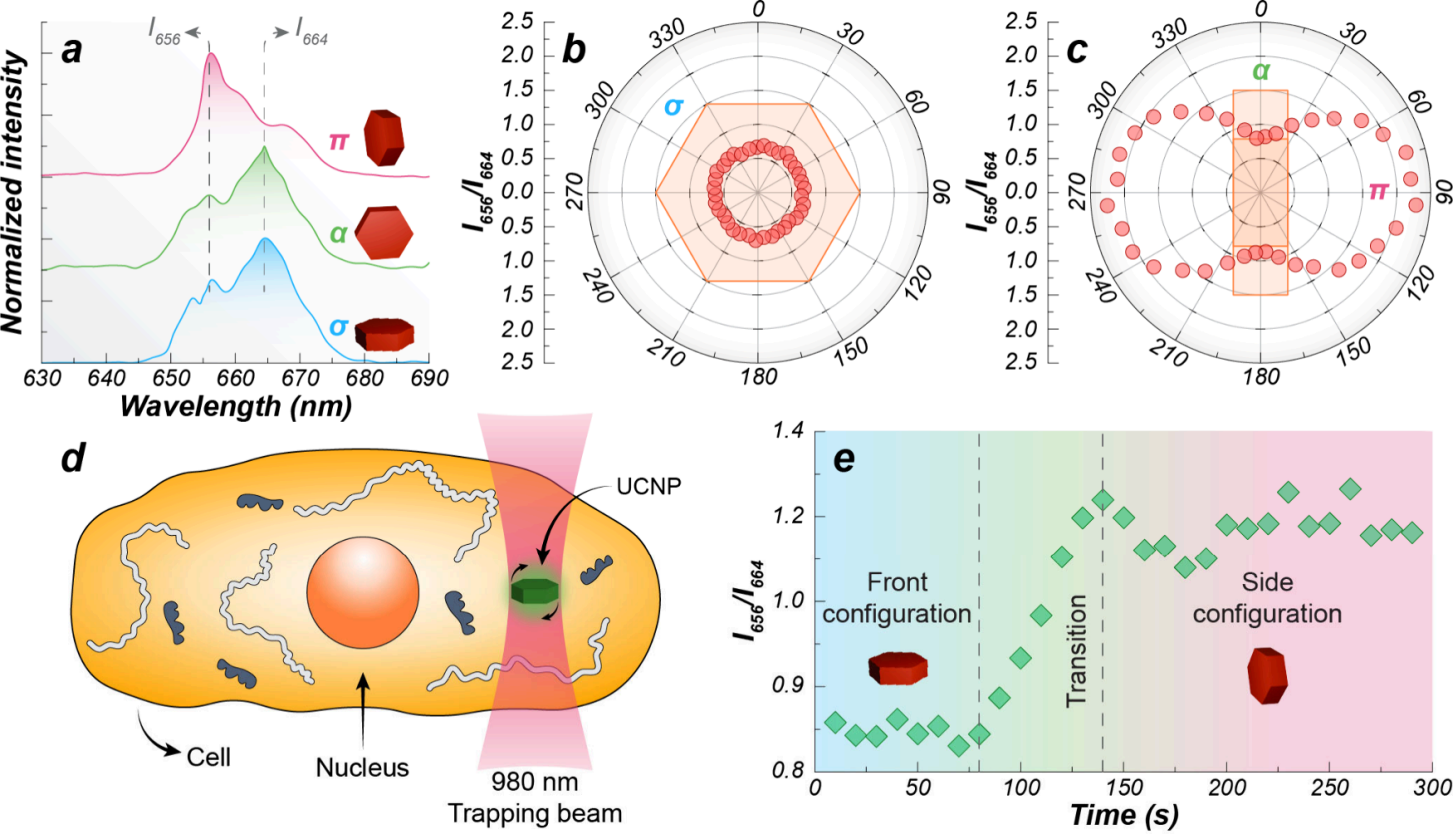
(a) Upconverting luminescence spectra (980 nm excitation) and the π, α, and σ polarization states. Polar diagrams of the intensity ratio *I*_656_/*I*_664_ as a function of the polarization angle for the (b) front (σ) and (c) side (α and π) configurations. (d) Schematic of a UCNP rotation inside a HeLa cell. (e) Evolution of the UCNP rotation inside a HeLa cancer cell. (a–c, e) Adapted from ref 4, with permission from the American Chemical Society, Copyright 2016.

A major challenge in luminescence sensing is cross-talk, where different stimuli, such as temperature, viscosity, or pressure, modify the same spectral features. In biological microrheology, simultaneous changes in temperature and viscosity, or laser-induced heating, can therefore bias mechanical readouts. To overcome this limitation, a recent contribution of our group demonstrated that a single rotating NaYF_4_:Yb^3+^/Er^3+^ microparticle can deliver decoupled measurements of temperature and viscosity.^79^ This strategy exploited the spectral and polarization anisotropies of Er^3+^ emission under 808 nm excitation. The green emission from thermally coupled levels served as a luminescent thermometer via the Boltzmann-based intensity ratio, while the polarization-sensitive red emission band (^4^F_9/2_ → ^4^I_15/2_) acted as a luminescence velocimeter. When the particle is rotated by circularly polarized light, the temporal evolution of the red-band ratio *R*_r_ = *I*_652_/*I*_665_ directly reports the angular speed Ω, which, combined with the known optical torque, yields the local viscosity (η) through the applied optical torque via Ω ∝ *P*/η.

This dual-channel strategy has been validated in scenarios directly relevant to microscale rheology. In a microfluidic mixing experiment, a rotating NaYF_4_:Yb^3+^/Er^3+^ particle reported a constant local temperature via *R*_r_, while its rotation slowed down as a more viscous polyacrylamide solution diffused into the chamber, allowing the viscosity increase to be tracked in real time solely from the red-band oscillations.^80^ In the second experiment, an additional 1450 nm heating beam was used to raise the local temperature of a polyacrylamide solution around the trapped particle; simultaneous analysis of the green and red bands yielded the temperature dependence of viscosity at the microscale, in good agreement with bulk viscometer data.

## 4 Conclusion

After more than 15 years working with lanthanide-doped UCNPs, we find them as compelling as when we first began studying them. While their optical behavior is better understood and their preparation has improved, many important questions remain open, particularly when UCNPs are used inside cells or studied individually. The contributions discussed in this Account highlight both the progress achieved and the challenges that persist.

In intracellular thermometry, UCNPs continue to provide a simple and reproducible method for temperature measurements at the submicrometric scale. Although their thermal sensitivity is not the highest among luminescent nanothermometers, the Boltzmann-based ratiometric approach is straightforward and compatible with standard microscopy, making UCNPs one of the most realistic options for real-time intracellular temperature tracking. However, the intracellular environment is complex and can influence the optical response underlying temperature readout. Factors such as pH, molecular crowding, viscosity, and cytoplasm-induced surface changes may alter UCNP luminescence. These effects are not yet fully understood and require dedicated investigation. Rather than comparing calibration curves obtained under different laboratory conditions, calibration experiments should be designed in the presence and absence of living processes. Progress will also depend on studies addressing long-term stability, intracellular surface modifications, and confinement within vesicles or other subcellular structures. Only then can UCNP-based intracellular thermometry be validated as a reliable quantitative method. In addition, the choice of excitation wavelength must be carefully evaluated. The widespread use of 980 nm excitation, which is absorbed by water, may induce local heating and lead to overestimated temperatures. Alternatively, nonabsorbing excitation wavelengths should therefore be explored.

Once internalized by cells, UCNPs are typically confined within lysosomes, where their close proximity may give rise to interparticle interactions. These interactions can significantly distort luminescence signals compared to those from isolated particles. Understanding such effects is essential for correctly interpreting intracellular fluorescence data. Our work has addressed this issue through the fabrication of hybrid nanostructures and through techniques enabling access to the luminescence of individual or a few UCNPs. Optical trapping studies revealed that radiative interactions between UCNPs are significant and can induce spectral distortions, which are often overlooked in biological studies. Single-particle optical trapping also exposed limitations. Trapping forces are weaker than commonly assumed, and stability decreases with increasing temperature. Higher dopant concentrations improve confinement but increase heating, creating an unresolved trade-off. Plasmonic strategies enhance trapping but induce excessive heating, prompting the need for alternative approaches such as dielectric metasurfaces.

Overall, while the optical properties and functional limits of UCNPs are now better defined, their behavior in complex environments remains incompletely understood. Continued progress will require systematic studies of intracellular effects, optical force–heating balance, and photonic strategies that enhance confinement without compromising thermal control.
